# New cooperative medical scheme decreased financial burden but expanded the gap of income-related inequity: evidence from three provinces in rural China

**DOI:** 10.1186/s12939-016-0361-5

**Published:** 2016-05-04

**Authors:** Jingdong Ma, Juan Xu, Zhiguo Zhang, Jing Wang

**Affiliations:** Department of Health Information, School of Medicine and Health Management, Tongji Medical College, Huazhong University of Science and Technology, Hubei, 430030 China; Department of Health Management, School of Medicine and Health Management, Tongji Medical College, Huazhong University of Science and Technology, Hubei, 430030 China; The Key Research Institute of Humanities and Social Science of Hubei Province, Huazhong University of Science and Technology, Hubei, 430030 China

**Keywords:** China, New Cooperative Medical Scheme, Financial burden, Inequity, Catastrophic health expenditure, Concentration index

## Abstract

**Background:**

Subsidizing healthcare costs through insurance schemes is crucial to overcome financial barriers to health care and to avoid high medical expenditures for patients in China. The health insurance could decrease financial risk by less out-of-pocket (OOP) payment, but not promise the protection equity. With the growth of New Cooperative Medical Scheme (NCMS) financing and coverage since 2008, the protection effectiveness and equity of the modified NCMS policies on financial burden should be further evaluated.

**Methods:**

A cross-sectional household survey was conducted in Zhejiang, Hubei, and Chongqing provinces by multi-stage stratified random sampling in 2011. A total of 1,525 households covered by the NCMS were analyzed. The protection effectiveness and protection equity of NCMS was analyzed by comparing the changes in health care utilization and medical expenditures, and the changes in the prevalence of catastrophic health expenditure (CHE) and its concentration indices (CIs) between pre- and post-NCMS reimbursement, respectively.

**Results:**

The medical financial burden was still remarkably high for the low income rural residents in China due to high OOP payment, even after NCMS reimbursement. In Hubei province, the OOP payment of the poorest quintile was almost same as their households’ annual expenditures. Even it was higher than their annual expenditures in Chongqing municipality. Effective reimbursement ratio of both outpatient and inpatient services were far lower than nominal reimbursement ratio originally designed by NCMS plans. After NCMS reimbursement, the prevalence of CHE was considerably high in all three provinces, and the absolute values of CIs were even higher than those before reimbursement, indicating the inequity exaggerated.

**Conclusion:**

Policymakers should further modify NCMS policy in rural China. The high OOP payment could be decreased by expanding the drug list and check directory for benefit package of NCMS to minimize the gap between nominal reimbursement ratio and effective reimbursement ratio. And the increase in medical expenditures should be controlled by monitoring excess demand from both medical service providers and patients, and changing fee-for-service payment for providers to a prospective payment system. Service accessibility and affordability for vulnerable rural residents should be protected by modifying regressive financing in NCMS, and by providing extra financial aid and reimbursement from government.

## Backgrounds

If taken as a major source of healthcare financing, out-of-pocket (OOP) payment, which is income-related, would cause disease-related financial burden for patients. Moreover, the OOP-based health financing was regressive, which made the poor pay a larger share of their incomes for OOP payment than the rich [1]. Russell S. emphasized that abandonment of needed care because of the limited payment capacity can lead to worse health, less productivity and income, and increased poverty [2]. Bowser et al. found that the heavy financial burden was caused not only by ill health but also by the inequitable distribution of disease-related financial burdens [3].

Subsidizing healthcare costs through insurance schemes is crucial to overcome financial barriers to health care and to avoid high medical expenditures [4]. Without an effective insurance coverage, households would have to rely on informal healthcare payment arrangements, such as using savings, borrowing money, selling assets, cutting food expenditures, or halting children from schooling [2]. So, universal health insurance coverage is regarded as a solution to alleviating disease-related financial burden.

The effectiveness of appropriate health insurance plans in financial protection has been well documented. In Turkey, risk pooling/health insurance was found to be beneficial and better protected people from catastrophic health expenditure (CHE) than other individuals with comparable income levels in other countries [5]. Laos [6] and Ireland [7] provided a universal health insurance through implementing new schemes or modifying existing policies, to decrease the population’s disease-related financial burden.

However, the reduction of OOP payment by health insurance corresponds to the contribution to the absolute decrease in financial burden, the equity of benefits from health insurance should be further investigated. Studies by Gwatkin D. and Wagstaff A. showed that the illness disproportionately afflicted the population and the poor were the most vulnerable populations because they would suffer a heavy financial burden, if health insurances failed to increase payments for the utilization of health services [8, 9]. Xu et al. emphasized that less households were likely affected by catastrophic payments only when OOP payment was reduced to levels lower than 15 % of the total medical expenditure [4]. Although the healthcare reform in Colombia was based on social insurance to reduce financial barriers to healthcare access and thus had shown an impact on OOP financing for the poor [10], health insurances could not ensure to decrease the regressivity. For instance, inequalities in OOP health care financing in Poland have decreased since the universal health care system was introduced in 1999, but they increased again in 2006 [1]. Furthermore, accessibility-related problems in lower income populations can persist even in countries with a universal health insurance coverage [11].

In China, New Cooperative Medical Scheme (NCMS), a voluntary insurance scheme designed for rural residents, was founded in 2003. The enrollment rate of NCMS gradually increased and has achieved 97.5 % in 2011. The scheme is co-financed by individuals, local government, and central government with a county as a risk pooling unit. The average annual flat-rate premium per capita of NCMS was 246 Chinese Yuan (¥) (US$37.14 based on the exchange rate on January 1, 2011) in 2011, including ¥200 ($30.20) from the government and ¥46 ($6.95) from rural residents. While in 2015, the flat-rate premium per capita of NCMS increased to 500 Chinese Yuan (¥) (US$80.65 based on the exchange rate on January 1, 2015) on average, including ¥380 ($61.29) from the government and ¥120 ($19.35) from rural residents.

Although reimbursement guidelines have been issued by the central government, the design and benefit packages of the NCMS in each county possibly differ. The NCMS plans in majority of the counties originally focused only on protecting against inpatient expenses with low ceiling, high deductible, and high co-insurance rates according to the limited funding pool [12, 13]. Thus, the effective reimbursement ratio (ERR) was very low in many counties. In particular, outpatient services were not initially covered by the NCMS. Wagstaff et al. found that the NCMS at that time took no effects on OOP payment, although it positively affected the utilization of health services between 2003 and 2005 [13] . In a field survey study, Sun et al. found that the OOP medical payments remained a burden for rural households in 2005 [14]. Yip et al. mentioned that the NCMS ignored to reduce the financial burden of outpatient medical expenditure of poor communities with chronic diseases [15]. Thus, the financial protection against high healthcare expenditures remained very restricted among the poor in rural China [13], because approximately 54 % of medical expenditures related to outpatient services for chronic disease had not been covered by the NCMS [16].

With the growth of NCMS financing and coverage since 2008, the modified NCMS policies engaged in enhancing the financial protection to achieve the objectives of the universal health insurance defined by the World Health Organization (WHO) [17]. The nominal reimbursement ratio (NRR) of inpatient services within the benefit packages had been set as 70 % in township hospitals, which were the lowest level institutions to provide inpatient services, since 2011. Furthermore, outpatient services have been generally covered with a NRR of 50 % in primary health institutions (including village clinics and township hospitals) in rural China since 2011, and the NRR of outpatient services for some chronic diseases, such as hemodialysis and aplastic anemia, was 70 %.

However, the protection effectiveness and protection equity of the NCMS plans on financial burden should be further evaluated. Although equity was not the main designed goal of health insurances, the needy deserve extra protection to avoid income-based inequity. Therefore, this study aimed to: 1) examine the protection effectiveness of NCMS under current polices by analyzing the health care utilization and medical expenditures, 2) investigate the protection fairness by comparing the changes in the prevalence of CHE and its concentration indices (CIs) among different income-level household groups, between pre- and post-NCMS reimbursement.

## Methods

### Sampling

A cross-sectional household survey was conducted in three provinces, Zhejiang, Hubei, and Chongqing directly under the central government, in 2011, which were in the Eastern, Central, and Western regions of China, respectively. Households were sampled by multi-stage stratified random sampling. Three counties in high-, medium-, and low-income levels were selected respectively from each province. Then, 2 towns from each county, 2 villages from each town, and 50 households from each village were randomly selected. The design samples comprised 1,800 households. Households in which some members did not participate in NCMS but in other medical insurance plans such as Urban Employee Basic Medical Insurance or Urban Resident Basic Medical Insurance were excluded from this study. In addition, the invalid questionnaires (loss of important information or with logical error) also were excluded. Finally a total of 1,525 questionnaires regarding households were qualified and analyzed in this study.

Information on socioeconomic characteristics, health care utilization, and medical expenditures of each member of the investigated households was collected by the slightly modified questionnaire used in the fourth National Health Services Survey in China 2008.

### Definition of key variables

OOP payment included all categories of health-related expenses paid directly by the household at the time the household received the health service, typically including doctors’ consultation fees, medication purchases, and hospital bills [18]. In this study, the OOP payment included the payment for co-insurance, co-payments, deductibles of outpatient services with a recall period of two weeks, those of inpatient care with a recall period of one year preceding the survey, and other payments for medicines, items and services not covered by insurance. However, the insurance premiums were excluded because this study focused on the measurement of the financial burden directly related to the illness.

CHE was defined as OOP payment exceeding a certain threshold in a given period, and threshold was calculated by medical expenditures sharing of household income or expenditure [19]. However, some households could achieve a consumption level higher than their income by mobilizing additional resources, such as borrowing money or selling assets [20]. The ability to pay, which was defined as the household expenditure that subtracted food expenditure from the total household consumption [4], was a more accurate indication of purchasing power than income reported in household surveys in many countries [18]. While in rural China, most residents are non-employed, and they obtain their food by self-production, the ability to pay was hard to accurately measure. Deaton et al. pointed out that in the territories that were characterized by substantial household-based production, lack of formal labor markets and high variability in household income across time, the value of household expenditure could be used as a proxy for permanent income and ability to pay [21]. So, in this article, the threshold of CHE was defined as OOP of a household over 40 % of the total household expenditure, according to the threshold of the WHO [18].

Concentration index (CI) measured the equitability of the distribution of an indicator across households with different incomes [22]. CI ranges from −1 to 1, with a value of 0 indicating a complete equality of the indicator across income groups. And the indicator was more equitable when CI was closer to 0. Negative CI values implied that the indicator was concentrated among households with low incomes, whereas positive values showed that the indicator was concentrated among households with high incomes.

Besides, variables that self-reported illness and doctor suggesting inpatient were used to reflect the health need of the surveyed households, and variables that outpatient utilization and inpatient utilization were used to reflect their health utilization. The detailed definition of each variable analyzed in this study and their abbreviations were set out in Table 1.

**Table 1:** Definitions and abbreviations of variables

Variable	Definition	Abbreviation	Variable type
Household number	Sampled household number	H-num	continuous
Household size	Number of household members	H-size	categorical
Household annual expenditure	The total expenditure of the whole household	H-expend	categorical
Household with aged members	Number of individuals aged 60 years and over per household	H-aged	categorical
Household education level	The highest level of education of any household member	Edu-level	categorical
Household vocation	Vocation of household head	H-voca	categorical
Self-reported illness	Frequency of self-reported illness in the past 14 days per household	Self-ill	continuous
Outpatient utilization	Frequency of accessing outpatient services in the past 14 days per household	Out-p	continuous
Doctor suggesting inpatient	Frequency of doctor suggesting inpatient services annually per household	Sug-inp	continuous
Inpatient utilization	Frequency of accessing inpatient services annually per household	In-p	continuous
Expenditures of OTC	Medical expenditures on over-the-counter medicine in the past 14 days per household	OTC-expend	continuous
Expenditures of outpatient	Medical expenditures on outpatient services in the past 14 days per household	Outp-expend	continuous
OOP of outpatient	OOP expenditures on outpatient services in the past 14 days per household	Outp-OOP	continuous
ERR of outpatient	ERR of outpatient services in the past 14 days per household	Outp-ERR	continuous
Expenditures of inpatient	Annual medical expenditures on inpatient services per household	Inp-expend	continuous
OOP of inpatient	Annual OOP expenditures on inpatient services per household	Inp-OOP	continuous
ERR of inpatient	ERR of inpatient services annually per household	Inp-ERR	continuous
Medical expenditures ratio	The ratio of medical expenditures sharing the annual household expenditures per household	Heal/Expend	continuous
OOP ratio	The ratio of OOP expenditures sharing the annual household expenditures per household	OOP/Expend	continuous

### Analysis

The sampled households in each province were classified respectively into quintiles from the poorest to the richest (Q1 to Q5) on the basis of the annual household income level. The differences in the household socioeconomic characteristics of each province were described among quintiles in each province.

Health care needs and utilization, medical expenditures and NCMS reimbursement for health services were compared among each income quintile to show the protection differences in NCMS. We calculated the CHE prevalence of each income quintiles pre- and post-reimbursement, and then compared their changes of each quintile. And the CIs of the CHE prevalence, pre- and post-reimbursement, were calculated to show equity change.

Continuous variables were analyzed by one-way ANOVA to compare the differences in means, and categorical variables were analyzed by Chi-square test to compare the differences in proportion. Data were coded and computerized using EpiData 3.0 and analyzed with SPSS 12.0 and STATA 13.0.

The Ethics Committee of Tongji Medical College, Huazhong University of Science and Technology approved the research proposal. All of the respondents agreed an informed consent before they were interviewed.

## Results

Table 2 showed the socioeconomic characteristics of the households in each quintile of the three provinces. All of the variables in each province, except H-aged in Hubei and Chongqing, were significantly different among the five income quintiles. There were fewer household members, lower annual expenditures, more aged members, less high level education and more farmers as vocation in the poorest quintile than other quintiles in the three provinces.

**Table 2:** Socioeconomic characteristics of income quintile in each province

Province	Socioeconomic characteristics	Poorest (Q1)	Q2	Q3	Q4	Richest (Q5)	Total	Sig.*
Hubei	H-num (N)	241	169	136	60	27	633	
	H-size (N(%))							
	1–3 members	153(63.49)	63(37.28)	39(28.68)	11(18.33)	3(11.11)	269(42.50)	χ^2^=81.178
	4 members and more	88(36.51)	106(62.72)	97(71.32)	49(81.67)	24(88.89)	364(57.50)	*p*<0.001
	H-expend (N(%))							
	Less than ¥10,000	207(85.89)	48(28.40)	19(13.97)	3(5.00)	1(3.70)	278(43.92)	χ^2^=415.619
	¥10,000-30,000	31(12.86)	121(71.60)	98(72.06)	34(56.67)	11(40.74)	295(46.60)	*p*<0.001
	¥30,000 and more	3(1.24)	0(0.00)	19(13.97)	23(38.33)	15(55.56)	60(9.48)	
	H-aged (N(%))							
	0	95(39.42)	72(42.60)	76(55.88)	28(46.67)	12(44.44)	283(44.71)	*χ* ^2^ = 14.492
	1	65(26.97)	50(29.59)	27(19.85)	12(20.00)	4(14.81)	158(24.96)	*p* = 0.070
	2 and more	81(33.61)	47(27.81)	33(24.26)	20(33.33)	11(40.74)	192(30.33)	
	Edu-level (N(%))							
	primary school	94(39.00)	22(13.02)	4(2.94)	1(1.67)	0(0.00)	121(19.12)	χ^2^=106.928
	further	147(61.00)	147(86.98)	132(97.06)	59(98.33)	27(100)	512(80.88)	*p*<0.001
	H-voca (N(%))							
	formal employee	6(2.49)	12(7.10)	11(8.09)	5(8.33)	1(3.70)	35(5.53)	χ^2^=20.655
	farmer	197(81.74)	120(71.01)	95(69.85)	35(58.33)	18(66.67)	465(73.46)	*p*=0.008
	informal employee	38(15.77)	37(21.89)	30(22.06)	20(33.33)	8(29.63)	133(21.01)	
Chongqing	H-num	155	184	88	33	38	498	
	H-size (N(%))							
	1–3 members	91(58.71)	67(36.41)	15(17.05)	9(27.27)	5(13.16)	187(37.55)	χ^2^=56.601
	4 members and more	64(41.29)	117(63.59)	73(82.95)	24(72.73)	33(86.84)	311(62.45)	*p*<0.001
	H-expend (N(%))							
	Less than ¥10,000	143(92.26)	36(19.57)	8(9.09)	3(9.09)	4(10.53)	194(38.96)	χ^2^=440.401
	¥10,000–30,000	11(7.10)	144(78.26)	70(79.55)	15(45.45)	9(23.68)	249(50.00)	*p*<0.001
	¥30,000 and more	1(0.65)	4(2.17)	10(11.36)	15(45.45)	25(65.79)	55(11.04)	
	H-aged (N(%))							
	0	46(29.68)	69(37.50)	36(40.91)	14(42.42)	15(39.47)	180(36.14)	*χ* ^2^ = 13.374
	1	42(27.10)	40(21.74)	29(32.95)	11(33.33)	8(21.05)	130(26.10)	*p* = 0.100
	2 and more	67(43.23)	75(40.76)	23(26.14)	8(24.24)	15(39.47)	188(37.75)	
	Edu-level (N(%))							
	primary school	73(47.10)	41(22.28)	8(9.09)	4(12.12)	4(10.53)	130(26.10)	χ^2^=58.133
	further	82(52.90)	143(77.72)	80(90.91)	29(87.88)	34(89.47)	368(73.90)	*p*<0.001
	H-voca (N(%))							
	formal employee	2(1.29)	6(3.26)	6(6.82)	2(6.06)	1(2.63)	17(3.41)	χ^2^=22.244
	farmer	125(80.65)	131(71.20)	55(62.50)	19(57.58)	20(52.63)	350(70.28)	*p*=0.004
	informal employee	28(18.06)	47(25.54)	27(30.68)	12(36.36)	17(44.74)	131(26.31)	
Zhejiang	H-num	79	82	85	89	59	394	
	H-size (N(%))							
	1-3 members	68(86.08)	43(52.44)	21(24.71)	23(25.84)	9(15.25)	164(41.62)	χ^2^=104.210
	4 members and more	11(13.92)	39(47.56)	64(75.29)	66(74.16)	50(84.75)	230(58.38)	*p*<0.001
	H-expend (N(%))							
	Less than ¥10,000	65(82.28)	13(15.85)	1(1.18)	0(0.00)	0(0.00)	79(20.05)	χ^2^=398.122
	¥10,000–30,000	9(11.39)	63(76.83)	70(82.35)	35(39.33)	5(8.47)	182(46.19)	*p*<0.001
	¥30,000 and more	6(6.33)	6(7.32)	14(16.47)	54(60.67)	54(91.53)	133(33.76)	
	H-aged (N(%))							
	0	11(13.92)	34(41.46)	37(43.53)	53(59.55)	24(40.68)	159(40.36)	χ^2^=40.031
	1	32(40.51)	18(21.95)	18(21.18)	18(20.22)	18(30.51)	104(26.40)	*p*<0.001
	2 and more	36(45.57)	30(36.59)	30(35.29)	18(20.22)	17(28.81)	131(33.25)	
	Edu-level (N(%))							
	primary school	62(78.48)	27(32.93)	17(20.00)	3(3.37)	2(3.39)	111(28.71)	χ^2^=147.491
	further	17(21.52)	55(67.07)	68(80.00)	86(96.63)	57(96.61)	283(71.83)	*p*<0.001
	H-voca (N(%))							
	formal employee	0(0.00)	7(8.54)	8(9.41)	11(12.36)	5(8.47)	31(7.87)	χ^2^=18.157
	farmer	58(73.42)	46(56.10)	46(54.12)	43(48.31)	28(47.46)	221(56.09)	*p*=0.020
	informal employee	21(26.58)	29(35.37)	31(36.47)	35(39.33)	26(44.07)	142(36.04)	

*α = 0.05

Table 3 showed the differences in health care needs and utilization among each quintile. There were no significant differences in all variables in the three provinces, except Out-p in Zhejiang. The frequency of outpatient service utilization was the highest in the richest quintile in Zhejiang, even though the disease prevalence, indicated by Self-ill, did not significantly differ among the quintiles.

**Table 3:** Health care need and utilization of each income quintile

Province	Health care need and utilization frequency	Poorest (Q1)	Q2	Q3	Q4	Richest (Q5)	Total	Sig.*
Hubei	Self-ill (mean)	0.83	0.78	0.83	0.80	0.59	0.80	*F* = 0.378
								*p* = 0.824
	Out-p (mean)	0.38	0.36	0.48	0.38	0.33	0.39	*F* = 0.707
								*p* = 0.587
	Sug-inp (mean)	0.88	0.62	0.53	0.53	0.26	0.67	*F* = 2.248
								*p* = 0.063
	In-p (mean)	0.39	0.43	0.33	0.35	0.26	0.38	*F* = 0.679
								*p* = 0.607
Chongqing	Self-ill (mean)	0.67	0.72	0.70	0.58	0.63	0.69	*F* = 0.386
								*p* = 0.819
	Out-p (mean)	0.30	0.25	0.35	0.21	0.24	0.28	*F* = 0.812
								*p* = 0.518
	Sug-inp (mean)	0.65	0.63	0.42	0.67	0.55	0.59	*F* = 0.829
								*p* = 0.507
	In-p (mean)	0.43	0.41	0.32	0.67	0.50	0.42	*F* = 1.810
								*p* = 0.125
Zhejiang	Self-ill (mean)	0.46	0.54	0.49	0.49	0.71	0.53	*F* = 1.078
								*p* = 0.367
	Out-p (mean)	0.11	0.16	0.12	0.16	0.37	0.17	*F*=3.122
								*p*=0.015
	Sug-inp (mean)	0.29	0.20	0.36	0.30	0.44	0.31	*F* = 1.829
								*p* = 0.122
	In-p (mean)	0.27	0.17	0.35	0.30	0.39	0.29	*F* = 1.815
								*p* = 0.125

*α = 0.05

Table 4 presents the medical expenditures and NCMS reimbursement of each quintile. Heal/Expend was significantly different among the quintiles in the three provinces, in particular, it was the highest in the poorest quintile but the lowest in the richest quintile. But OOP/Expend was significantly different among the quintiles only in Chongqing, which also was the highest in the poorest quintile but the lowest in the richest quintile. And Outp-expend and Inp-ERR were significantly different among the quintiles only in Zhejiang. Outp-expend was the highest in the richest quintile but the lowest in the poorest quintile, while Inp-ERR was the highest in the poorest quintile but the lowest in the middle quintile.

**Table 4:** Health care expenditure and NCMS reimbursement of each income quintile

Province	Health care expenditure	Poorest (Q1)	Q2	Q3	Q4	Richest (Q5)	Total	Sig.*
Hubei	OTC-expend (¥) (mean)	27.76	41.70	23.58	44.83	46.22	32.99	*F* = 0.988
								*p* = 0.413
	Outp-expend (¥) (mean)	228.59	200.89	310.85	171.92	77.70	227.06	*F* = 1.154
								*p* = 0.330
	Outp-OOP (¥) (mean)	177.31	168.13	271.71	153.05	73.04	188.39	*F* = 0.955
								*p* = 0.432
	Outp-ERR (%) (mean)	0.15	0.19	0.15	0.08	0.16	0.16	*F* = 0.327
								*p* = 0.859
	Inp-expend (¥) (mean)	4501.58	2931.42	3600.59	2522.50	3025.93	3638.26	*F* = 0.317
								*p* = 0.867
	Inp-OOP (¥) (mean)	3491.86	1859.15	2388.59	1822.83	1980.37	2596.25	*F* = 0.490
								*p* = 0.743
	Inp-ERR (%) (mean)	0.39	0.44	0.41	0.35	0.42	0.40	*F* = 0.371
								*p* = 0.829
	Heal/Expend (%) (mean)	422.60	108.10	69.52	44.49	19.06	209.72	*F*=2.417
								*p*=0.048
	OOP/Expend (%) (mean)	97.05	92.29	57.94	37.26	13.41	78.14	*F* = 0.033
								*p* = 0.998
Chongqing	OTC-expend (¥) (mean)	22.65	29.27	56.04	12.30	25.71	30.54	*F* = 2.320
								*p* = 0.056
	Outp-expend (¥) (mean)	136.55	117.89	189.66	44.24	82.79	128.82	*F* = 0.995
								*p* = 0.410
	Outp-OOP (¥) (mean)	125.73	95.31	152.11	32.24	80.18	109.48	*F* = 0.827
								*p* = 0.508
	Outp-ERR (%) (mean)	0.12	0.18	0.18	0.16	0.11	0.15	*F* = 0.415
								*p* = 0.797
	Inp-expend (¥) (mean)	2665.23	2170.43	2885.34	2950.00	1590.26	2458.15	*F* = 0.372
								*p* = 0.828
	Inp-OOP (¥) (mean)	1757.00	1527.72	2346.14	2184.85	1072.50	1752.51	*F* = 0.466
								*p* = 0.761
	Inp-ERR (%) (mean)	0.37	0.36	0.32	0.46	0.37	0.37	*F* = 0.587
								*p* = 0.672
	Heal/Expend (%) (mean)	178.39	46.78	41.22	26.15	17.84	83.18	*F*=5.445
								*p*<0.001
	OOP/Expend (%) (mean)	148.51	37.04	33.88	15.80	15.76	68.14	*F*=5.371
								*p*<0.001
Zhejiang	OTC-expend (¥) (mean)	44.18	55.85	39.41	47.35	57.51	48.29	*F* = 0.222
								*p* = 0.926
	Outp-expend (¥) (mean)	35.70	136.77	61.76	129.55	270.00	118.64	*F*=3.230
								*p*=0.013
	Outp-OOP (¥) (mean)	30.76	122.74	52.00	124.16	204.71	101.63	*F* = 2.248
								*p* = 0.063
	Outp-ERR (%) (mean)	0.22	0.08	0.12	0.10	0.21	0.14	*F* = 0.787
								*p* = 0.539
	Inp-expend (¥) (mean)	3950.00	3251.22	2865.88	2284.83	2683.05	3004.82	*F* = 0.254
								*p* = 0.907
	Inp-OOP (¥) (mean)	2227.22	2394.51	2154.12	1652.25	1762.71	2046.83	*F* = 0.104
								*p* = 0.981
	Inp-ERR (%) (mean)	0.46	0.35	0.19	0.21	0.27	0.28	*F*=4.515
								*p*=0.002
	Heal/Expend (%) (mean)	95.98	49.46	21.74	21.56	27.19	43.17	*F*=2.595
								*p*=0.036
	OOP/Expend (%) (mean)	64.54	41.91	18.23	19.70	21.54	33.27	*F* = 1.884
								*p* = 0.112

*α = 0.05

The most evident gap between Heal/Expend and OOP/Expend was found in the poorest quintile of Hubei, and the ratio of medical expenditures to household expenditures was reduced to 325.55 % after NCMS reimbursement. But even so, OOP payment of the poorest quintile was almost the same as those households’ annual expenditures in Hubei, or it was more than households’ annual expenditures in Chongqing, after medical expenditures were reimbursed by NCMS. Outp-ERR of most quintiles in the three provinces was less than 20 %, and Inp-ERR of most quintiles was less than 40 %. Both were far lower than the designed NRR of NCMS plans in 2011.

Table 5 illustrates the prevalence of CHE and CIs pre- and post-NCMS reimbursement. The prevalence of CHE was significantly different among the quintiles in the three provinces, whether pre- or post-reimbursement. Although the obvious change of the CHE prevalence between pre- and post-reimbursement occurred in the poorest or poorer quintile of each province, the prevalence of CHE was still considerably high in each quintile even after NCMS reimbursement. For instance, the prevalence of CHE was still 31.12 % in the overall Hubei sample. The CIs of CHE prevalence, whether pre- or post-NCMS reimbursement, were negative and close to 0 in the three provinces. However, the absolute values of CIs after NCMS reimbursement were even more than those before reimbursement in the three provinces, which implied the inequity exaggerated.

**Table 5:** Prevalence of CHE and CIs before and after NCMS reimbursement

Province	Prevalence of CHE	Poorest (Q1)	Q2	Q3	Q4	Richest (Q5)	Total	Sig.*	Concentration index(CI)
Hubei	Pre-reimbursement(N (%))	100(41.49 %)	60(35.50 %)	44(32.35 %)	16(26.67 %)	4(14.81 %)	224(35.39 %)	χ^2^=11.472	−0.0930
								*p*=0.022	
	Post-reimbursement(N (%))	93(38.59 %)	50(29.59 %)	38(27.94 %)	13(21.67 %)	3(11.11 %)	197(31.12 %)	χ^2^=14.643	−0.1190
								*p*=0.006	
	Change(Pre-Post)	7(2.90 %)	10(5.91 %)	6(4.41 %)	3(5.00 %)	1(3.70 %)	27(4.27 %)	----	0.0260
Chongqing	Pre-reimbursement(N (%))	74(47.74 %)	49(26.63 %)	33(25.00 %)	3(9.09 %)	3(7.89 %)	151(30.32 %)	χ^2^=40.717	−0.2279
								*p*<0.001	
	Post-reimbursement(N (%))	69(44.52 %)	41(22.28 %)	19(21.59 %)	3(9.09 %)	2(5.26 %)	134(26.91 %)	χ^2^=42.080	−0.2495
								*p*<0.001	
	Change(Pre-Post)	5(3.22 %)	8(4.35 %)	14(3.41 %)	0(0.00 %)	1(2.63 %)	17(3.41 %)	----	0.0216
Zhejiang	Pre-reimbursement(N (%))	27(34.18 %)	21(25.61 %)	17(20.00 %)	12(13.48 %)	12(20.34 %)	89(22.59 %)	χ^2^=11.212	−0.1498
								*p*=0.024	
	Post-reimbursement(N (%))	24(30.38 %)	16(19.51 %)	12(14.12 %)	8(8.99 %)	10(16.95 %)	70(17.77 %)	χ^2^=14.269	−0.1821
								*p*=0.006	
	Change(Pre-Post)	3(3.80 %)	5(6.10 %)	5(5.88 %)	4(4.49 %)	2(3.36 %)	19(4.82 %)	----	0.0323

*α = 0.05

## Discussion

The results of our study indicate the protection of NCMS under current polices have taken some effects, because the OOP payment was much less than the total medical expenses. But the protection effectiveness was weak, for OOP/Expend, a common indicator of financial burden, was still extremely high, even after the NCMS reimbursement for the outpatient and inpatient expenditures. And the risk of CHE was still high in most of households, for the prevalence of CHE was over 15 % in more than 3 quintiles of each province. These findings were aligned to a system review by Liang X. et al. who declared no clear evidence supported that NCMS could decrease the catastrophic health expenditure in Chinese rural population [23]. To make it worse, NCMS did not provide fair financial protection, according to the change of the CIs of the CHE prevalence. In addition, the availability of the care among the poor was less than the richer, so remarkably high burden expanded the primary inequity.

One reason leading to high OOP payment could be the considerable gap existing between NRR and ERR, mainly because of the limited benefit package that many drugs or medical checks were uncovered. So, the ERR of both outpatients and inpatients was far lower than the promised NRR. Moreover, household spending on outpatient services and drugs rather than on hospitalization could be the primary contributor to medical impoverishment according to Wagstaff’s report [24]. Thus, insufficient outpatient reimbursement, reflected by the lower NRR of outpatient than that of inpatient, in NCMS could be an important reason causing high OOP payment, especially in low-income households. It have been testified in our previous study which indicated that high deductible for outpatient services creates a financial burden for households containing people with chronic diseases [25].

Unreasonable increasing in health expenses aggravated the medical financial burden of low-income households. According to Hu et al., fees that households in China paid to access services nowadays were 18 times greater than those in 1990 [26]. Liu Y. et al. and Wagstaff A. et al. all found that the increasing health expense could drive many families into poverty or sink them even further into poverty, despite having health reimbursement [24, 27]. So, Li Y. et al. recommended an upgrade of benefit packages was needed and effective cost control mechanisms on the provider side should be considered, because higher prevalence of catastrophic health expenditure and medical impoverishment correlates to increased health care need, even after NCMS reimbursement [28].

Apart from medical expenditure augmenting due to price increase, services have been excessively delivered to patients by health providers because of fee-for-service payment mechanism. Meng Q. et al. reported that rapid increases in health-care use and in demand (possibly supplier-induced) have caused an escalation of medical expenditures in a way that threatens the sustainability of the scheme [29]. While payment reform on provider side, such as pay-for-performance, was testified to be effective to reduce antibiotic prescriptions and total spending per visit to community health clinics according to Yie W. et al. [30].

Meanwhile, insurance may prompt the insured individuals to obtain more expensive care than their needs, and result in increasing levels of OOP spending, which had already happened in China’s urban insurance scheme [5]. Zhang Y. et al. have illustrated that patients tend to exaggerate their health needs to seek expensive inpatient services instead of staying in the outpatient department, because reimbursement for outpatient services was much less than inpatient in NCMS [31]. In our study, the richest households rather than the poor ones have more outpatient services times, with a prerequisite that no significant difference of self-reported illness times. Whitehead et al. revealed that the phenomena of irrational drug use and prescription in low-income countries were associated with large OOP drug payments and led to the medical poverty trap [16].

Moreover, high OOP payment could exacerbate the weak financial protection of NCMS, because the equity measure (i.e. the CIs) got worse after NCMS reimbursement based on our findings. Yang W. also reported that OOP payment was concentrated disproportionately among the poor even after NCMS reimbursement [32], and income-related inequity in access to health care was not decreased after the expansion of NCMS across the rural China [33]. Van Minh et al. demonstrated that the high proportion of household direct OOP payment reflected an inequity issue in a health system in general, because the amount of OOP payment can be highly related to income [34]. In our study, the poor households were more vulnerable than the others, because they had fewer household members, less annual income, more aged members, less education and higher proportion of farmers. In addition, the level of OOP payment sharing of annual household expenditures greater than 40 % can result in inequity in health care, according to WHO [5]. In fact, OOP/Expend of Hubei and Chongqing was 78.14 and 68.14 % (see Table 4) respectively in our study. So, high OOP payment would reduce the health services affordability of the needy population, and decrease the protection equity finally.

Another reason that caused inequitable protection in NCMS could be inequity in financing. The universal coverage should be financed on the basis of the ability to pay, and the benefits received should be based on the needs for health care in health systems [30]. Income-related financing of health insurance has been widely used in many countries, even for the informal employee sector, such as in South Africa [30] and Mexico [35]. However, households had to pay a flat-rate premium for NCMS in rural China, which meant all of the households, regardless of variant income levels, paid the same premiums within a county. Therefore, regressive financing in NCMS made the poor households bear heavier burden than the rich, and the rich, who have spare money, were urged to avail more health services than what they actually need.

This study is characterized by several limitations. First, the estimations were based on self-reported illnesses and the corresponding costs rather than on those diagnosed by a physician and expense information system. Thus, the information may be either over- or under-reported. However, the questions used to describe the self-reporting of illnesses and the corresponding costs were obtained from the National Health Services Survey, which has been validated nationally. Second, the protection of NCMS was underestimated because the OOP payment sharing of annual household expenditures would be exaggerated compared with a unit of individual when the protection was calculated as a unit of household. In terms of the smallest risk-sharing unit, the calculation based on the household unit was much closer to the actual status of coping with medical financial burden. Third, three provinces sampled specifically could only reflect limited effectiveness of NCMS in China. And we believed that the actual NCMS protection level of rural residents had got further promoted since 2011, with NCMS reform keeping implemented.

## Conclusion

Although modified NCMS policies could reduce medical financial burden for rural residents to some extent, the protection effectiveness was still limited, and the result of NCMS reimbursement aggravated health inequity. Firstly, policymakers should decrease the high OOP payment by expanding the benefit package with expanding the drug list and check directory of NCMS to minimize the gap between NRR and ERR. Then, the increasing of medical expenditures should be controlled by monitoring excess demand from both providers and patients, and modifying fee-for-service payment for providers to a prospective payment system. Service accessibility and affordability of vulnerable rural residents should be protected by changing regressive financing in NCMS, and by providing extra aid and reimbursement from government.

Lessons from three provinces in China provided evidence that the modified health insurance plans could not guarantee financial protection effectiveness and equity. Periodic monitor and assessment could help adjust such policies timely to achieve the designed aims.

## Abbreviations

CHE: health spending and catastrophic health expenditure
CI: Concentration index
CIs: concentration indices
ERR: effective reimbursement ratio
NCMS: New Cooperative Medical Scheme
NRR: nominal reimbursement ratio
OOP: out-of-pocket
WHO: World Health Organization
